# Crosado embalming related alterations in the morpho-mechanics of collagen rich tissues

**DOI:** 10.1038/s41598-025-90378-5

**Published:** 2025-02-24

**Authors:** Joanna Tomlinson, Mario Scholze, Benjamin Ondruschka, Niels Hammer, Johann Zwirner

**Affiliations:** 1 School of Anatomy, University of Bristol, Bristol, UK; 2 Department of Anatomy, University of Otago, Dunedin, New Zealand; 3 Institute of Materials Science and Engineering, Chemnitz University of Technology, Chemnitz, Germany; 4 Institute of Legal Medicine, University Medical Center Hamburg-Eppendorf, Hamburg, Germany; 5 Department of Macroscopic and Clinical Anatomy, Medical University of Graz, Graz, Austria; 6 Department of Orthopaedic and Trauma Surgery, University of Leipzig, Leipzig, Germany; 7 Fraunhofer IWU, Dresden, Germany; 8 Department of Oral Sciences, University of Otago, Dunedin, New Zealand

**Keywords:** Biomechanical property, Dura mater, Embalming, Extra-cellular matrix, Iliotibial band, Phenoxyethanol, Water content, Anatomy, Musculoskeletal system, Ligaments, Tendons, Biomaterials, Soft materials

## Abstract

Crosado-embalming has been successfully used as embalming technique in research and teaching for over 20 years. It is applied in biomechanical testing experiments if the fresh tissues are unavailable, e.g., for cultural, ethical, logistical or health and safety reasons. However, features of human Crosado-embalmed tissues biomechanical characteristics including its load-deformation properties in comparison to fresh tissues and its controllability through hydration fluids may be insightful and therefore need to be studied further. This study compared the uniaxial load-deformation properties and the cross-sectional area (CSA) measurements of fresh-frozen and Crosado-embalmed collagen-rich tissues, namely the iliotibial band (ITB, 16 unembalmed and 35 embalmed specimens) and cranial dura mater (DM, 60 unembalmed cadavers, and 25 embalmed specimens). The water content of 120 Crosado-embalmed ITB samples (30 cadavers) were analysed considering established rehydration treatments, including polyethylene glycol (PEG). Crosado-embalmed tissues presented an increased elastic modulus (EM) (all *p* < 0.050; e.g., Crosado ITB PEG only 306 ± 91 MPa vs. fresh-frozen ITB PEG only 108 ± 31 MPa; mean ± standard deviation; *p* < 0.001) and ultimate tensile strength (UTS) (e.g., Crosado ITB PEG only 46 ± 15 MPa vs. fresh-frozen ITB PEG only 21 ± 8 MPa; *p* < 0.001) when rehydrated similar to the fresh tissues. The maximum force was different for the dura mater (Crosado 25 ± 13 N vs. fresh 21 ± 20 N; mean ± standard deviation; *p* = 0.050) but not for the ITB. The CSA following rehydration in PEG only was decreased for Crosado-embalmed samples (3.4 ± 1.2mm^2^, ITB; 1.1 ± 0.5 mm^2^, DM) compared to fresh-frozen (5.8 ± 2.1mm^2^, ITB; 3.1 ± 1.2mm^2^, DM) (all *p* ≤ 0.003). Furthermore, rehydration effects were observed following 24 h of PEG treatment (untreated tissues, 49 ± 9% vs. PEG only, 77 ± 4%; *p* < 0.001), in comparison to fresh samples (69%) tissues were hyperhydrated. In conclusion, Crosado-embalming appears to alter collagen-rich tissues’ morphological and mechanical properties. While an increase in material properties of Crosado-embalmed tissues was observed (Emod and UTS), the overall load-bearing capacity and peak structural strength remained unaltered for ITB tissues. This may result from CSA-related, geometric or molecular alterations after the fixative and osmotic water protocols related to changes in the collagen backbone and water-binding capacity.

## Introduction

The biomechanical properties of human tissues are essential for both computational and physical^1,2^ models to understand joint kinematics and reactions to traumatic events, such as head injuries. As the availability of chemically unaltered (“fresh”) human tissue is scarce in certain countries for various reasons, such as due to limitations related to ethics, logistics, experience, and limited donations. Embalmed tissue is sometimes used instead for biomechanical testing purposes^3–10^. Each embalming/preservation method has different components, benefits, and limitations (Table 1).

**Table 1:** Summary of different embalming solutions used in biomechanics testing based on published information with comments on ligament structure where available^11–20^.

Preservation method	Ethanol-based	Formaldehyde-based	Crosado	Thiel	Fresh-frozen
Contents	Various mixtures including ethanol	Various mixtures including formaldehyde	Ethanol, formaldehyde, glycerine, phenoxyethanol and water in the initial fixation solution^11^.	Various mixtures including: ethanol, ammonium nitrate, boric acid, formaldehyde, chlorocresol, propylene glycol, morpholine, potassium nitrate, sodium sulphite^12^	No additional solutions
Impact on soft tissues					
Biomechanical properties	Stiffens tissue^13^	Stiffens tissue^13^	Not investigated	Stiffens^12,14–17^ and softens tissue^18^	Considered life-like
Degradation	None known	None known, stronger disinfecting ability than Thiel^19^	None known	Adipose degradation, discolouration in contact with air^12^	Over time and exacerbated with presence of bacteria
Gross tissue structure	Ligaments considered bleached, dehydrated, stiffened^11^	Ligaments considered bleached, severely stiffened^11^	Ligaments considered welled, pliable^11^	Considered “life-like”^12^ Ligaments considered unaltered in appearance, and slightly softened^11^	Considered life-like^11^
Histological tissue structure	Well suited for histology^11^	Well suited for histology^11^	Well suited for histology^11^	Unsuitable for histology. Smaller muscle cells, fibre fragmentation and lack of nuclei^20^	Considered suitable for histology, dependant on days since death

These embalming effects alter different tissues in a variable manner. Crosado-embalming offers a potential alternative to other embalming techniques, with the aim to improve safety through the use of lower levels of formaldehyde compared to traditional formalin embalming^21^. However, Crosado-embalmed tissues have not been biomechanically compared to fresh tissues using standard tissue testing procedures, to date. The iliotibial band (ITB) and dura mater (DM) are reasonably well characterized in the fresh state and therefore are suitable model tissues to further investigate the biomechanical effects of the Crosado fixative on collagen-rich human tissues^5–10,22,23^.

Preparative procedures, such as sample rinsing, appear indispensable in restoring biomechanical properties of embalmed tissues closer to the fresh biomechanical function of the tissues. For example, formaldehyde- and ethanol-embalmed ITB tissues were shown to soften after rinsing in tap water^13^, likely due to rehydration effects. The elastic modulus (E_mod_) of Thiel-embalmed ITB and DM is unknown, but the E_mod_ of the flexor digitorum profundus tendons was equivalent to fresh samples when treating the samples with saline solution before and during the test^24^. However, the influence of rinsing on Crosado-embalmed samples in terms of their biomechanical response remains unknown.

To assure a high level of biomechanical property consistency, osmotically adapting is a routine step used in the literature for fresh tissues to reach a similar hydration state of all samples^23,25^, and therefore is often utilised for embalmed samples prior to biomechanical testing^23,25^. Several biomechanical properties of soft tissues are dependent on hydration^26^, thus avoiding both hyper- and hypohydration results in more homogeneous data sets. Commonly, the osmotic stress protocol involves submerging the samples within a dialysis membrane in a polyethylene glycol (PEG) solution of a defined concentration for a certain amount of time prior to the test^27^. This process should be individually established for each tissue type^27^. For the fresh human ITB and DM, immersion in PEG solution with a concentration of 2.5%^28^ and 5%^23,25^ for 24 h is considered to provide the most natively representative results. For Crosado-embalmed samples, an additional rinsing step in normal saline solution (0.9% sodium chloride) is considered beneficial in attempt to wash out the excess stiffening components, such as ethanol and glycerine^29^, thereby recovering the tissue as close as possible to its native mechanical behaviour. Furthermore, the water content of Crosado-embalmed samples including the influence of fluids used for rinsing and osmotic adaptation should be analysed together with the cross-sectional area (CSA) of the mechanically tested samples to determine the impact on the geometry of such tissues. Mechanically, the CSA forms a key parameter to calculate E_mod_ or ultimate tensile strength (UTS) and therefore even small changes may have a critical influence on the overall results in tissue biomechanics.

The primary aims of this study were:

1. To determine the biomechanical properties of Crosado-embalmed collagen-rich samples using ITB and DM as model tissues.
2. To compare the Crosado biomechanical properties to fresh samples from the same regions.

The secondary aims of the study were to determine:

1. The water content of Crosado-embalmed samples following various pre-testing conditions, such as immersion in saline, PEG, or both.
2. The CSA of the samples.
3. If the CSA could be an indicator of the water content of the sample.

## Materials and methods

The methods were approved by the institutions University of Leipzig (ref: 486/16-ek) and University of Otago (ref: H20/071) as part of the ethical application, these were performed in accordance with health and safety and other relevant guidelines. For German tissues, in line with the Saxonian Death and Funeral Act of 1994 and German law, the state attorney as the legal representative was informed about the use of the tissues for research purposes in each case. Written and informed consent was obtained from the body donors, while being alive, for their post mortem donation for teaching and research purposes in conjunction with the New Zealand Human Tissue Act (2008). Furthermore, Māori consensus from the Ngāi Tahu Research Consultation Committee was sought and provided. The tissues used for this given trial were divided into specific subgroups; those for mechanical testing and those for water content analysis.

### Tissue preparation for mechanical testing

Fresh human ITB (*n* = 16, taken from 16 individuals, mean age ± standard deviation: 50 ± 23 years; 5 females, 11 males) and DM samples from the brain at regions without blood vessels (*n* = 60; taken from 60 individuals, 51 ± 22 years; 22 females, 38 males) were retrieved during forensic dissections from semi-randomly allocated regions throughout the tissue, i.e., tissues from the proximal, central and distal portion of the ITB were randomly selected and distributed evenly between groups., Additionally, tissue samples from the ITB (*n* = 35; taken from 35 individuals; 82 ± 11 years; 16 females, 19 males) and DM (*n* = 25; taken from 25 individuals, 81 ± 9 years; 11 females, 14 males) of similar thickness were retrieved from Crosado-embalmed bodies^21^ prior to specimen collection and in accordance with national/ international/ institutional guidelines. Tissues were consistently retrieved to ensure that collagen fibres were orientated along the gauge length of the sample. This later resulted in stretching the collagen bundles along their length during tensile testing (parallel to the lateral edges of the tissues along the longer edge). Tissues that were recorded as or visibly altered by degradation, musculoskeletal degeneration, or any kind of pathology, were excluded. Prior to water content adaptation and mechanical processing, the tissues were cut into dumbbell shapes with a width of 5 mm and a gauge length of 10 mm using a customized template^30,31^.

### Water content adaptation

**Fig. 1:**
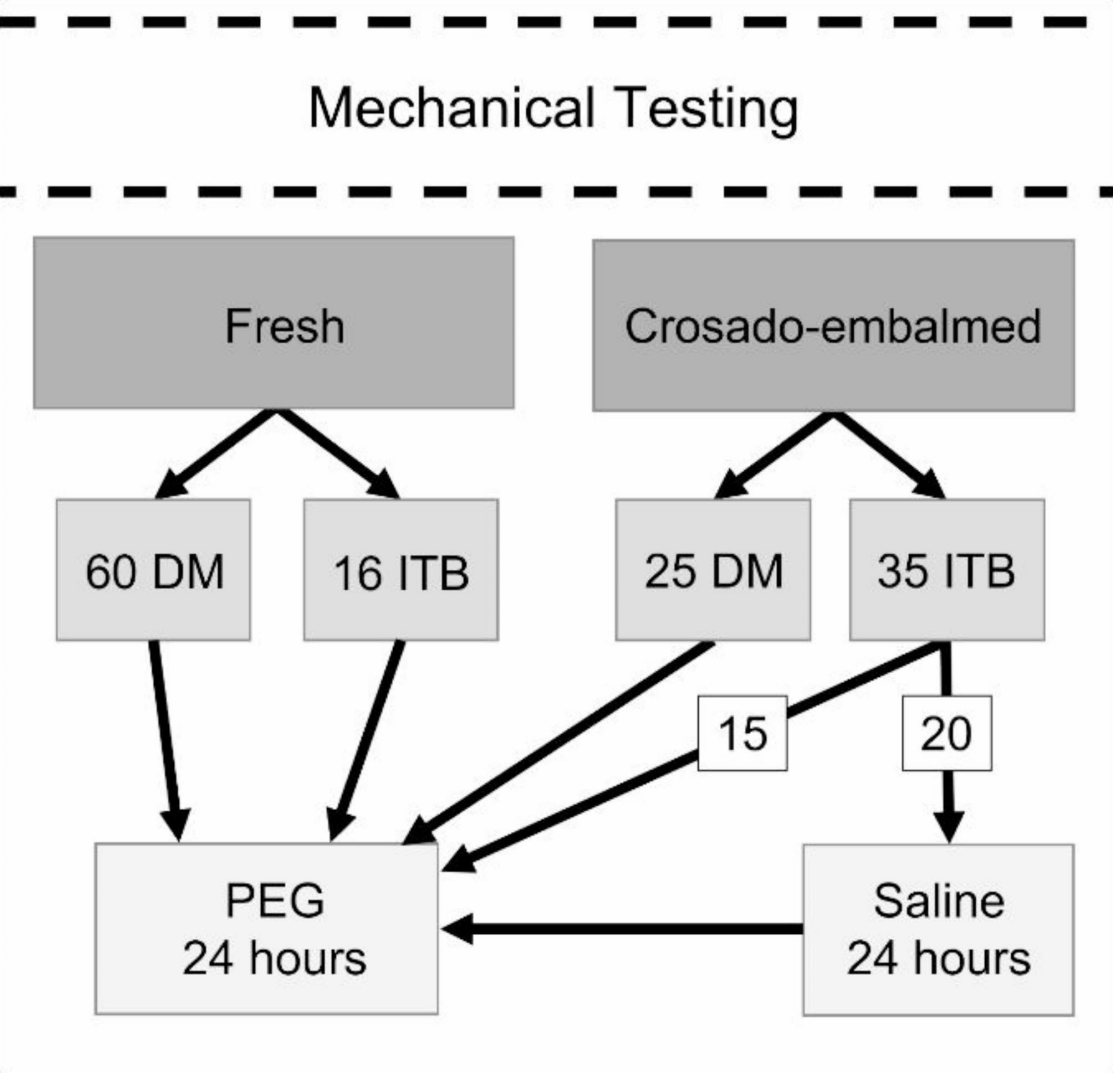
Diagram of groups for mechanical testing.

For the PEG only - groups, all fresh samples, Crosado-embalmed DM samples and 15 of the Crosado-embalmed ITB samples (82 ± 8 years; 5 females, 10 males) were placed into dialysis membranes (Spectra/ Por^®^; molecular weight cut off 6 to 8 kDa) and submerged into buffered PEG (Merck KGaA, Darmstadt, Germany; molecular weight 20 kDa; pH 7) for 24 h as done before^22,23^ with a concentration of 2.5%^28^ and 5%^23,25^, for ITB and DM, respectively (Fig. 1). The remaining 20 Crosado-embalmed ITB samples (85 ± 9; 6 females, 14 males) were rinsed in saline solution (0.9% sodium chloride) for 24 h before being treated with PEG (“Saline then PEG”).

### Mechanical testing

Immediately prior to the mechanical tests, the CSAs of the dumbbells were determined as follows: they were moulded using polysiloxane impression material (medium-bodied, Exahiflex; GC Corporation, Tokyo, Japan), these casts were then removed and sectioned into two halves (perpendicular to the gauge length), then digitized with a commercial scanner (Perfection 7V750Pro; Seiko Epson Corporation, Suwa, Japan; 1200 dpi) using a reference scale. The software Measure 2.1d (DatInf GmbH, Tübingen, Germany) was used after digitalizing the casts, as one observer calculated the area of the tissue, and then calculated the mean of the two analysed sides in accordance with standardised practices^30,31^.

**Fig. 2:**
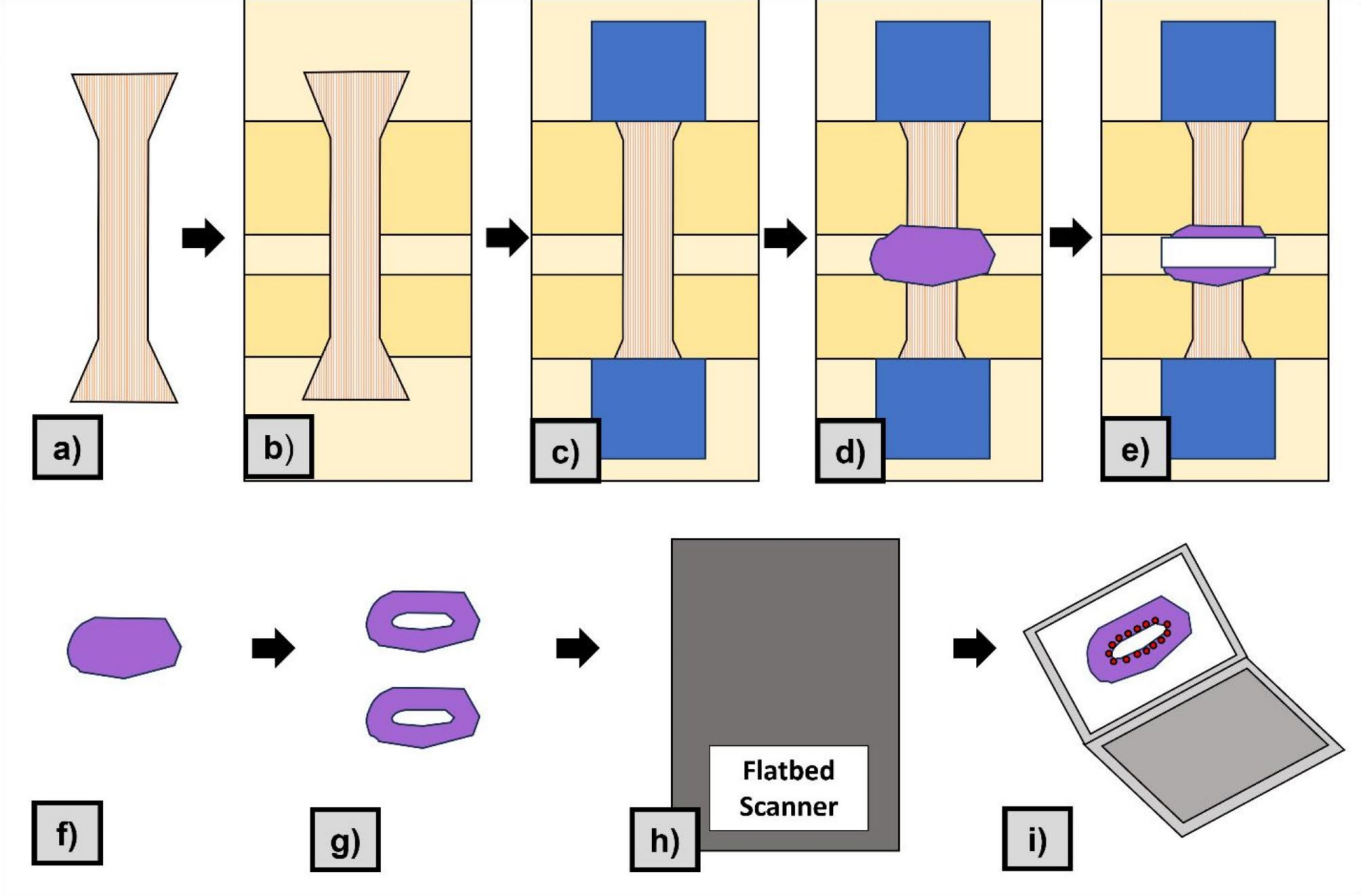
Schematic representation of the process to obtain measurement of the cross-sectional areas. (**a**) Tissue cut into dumbbell shape using template. (**b**) Tissue placed on mount for clamping (yellow rectangle). (**c**) Custom clamps (blue square) placed around tissue at fixed points. (**d**) Polysiloxane impression material (purple shape) placed around central portion of the tissue. (**e**) Moulding clamps (white rectangle) placed around polysiloxane impression material to ensure good coupling between tissue and impression material, and left to harden. (**f**) Hardened impression material is removed (the sample is ready for testing). (**g**) Using a scalpel, polysiloxane impression material sectioned in half to visualised cross-section created by tissue. (**h**) Sectioned impressions scanned on flatbed scanner. (**i**) The cross section was traced on computer software to quantify the cross-sectional area.

A universal testing machine (AllroundLine Z020 with an Xforce P load cell of 2.5 kN ( ISO 7500 accuracy grade 1; accuracy < 1%, repeatability < 1%, reversibility < 1.5%, zero error < 1%, resolution < 0.5% - all five criteria are fulfilled from 0.4% of the maximum force, which corresponds to 10 N) and testControl II measurement electronics; all Zwick Roell, Ulm, Germany) was used for uniaxial tensile testing at room temperature (22 °C). Customized 3D-printed polylactic acid clamps with an interlocking pyramidal pattern were used to prevent sample slippage and dehydration of the tested samples by enabling quick handling^30,31^. A stochastic graphite speckle pattern, which is known not to bias the biomechanical properties^22^, was applied to the surface of each tested sample allowing strain measurement during mechanical testing. The samples were preconditioned with 20 load-unload cycles up to 10% of the expected maximum force (F_max_) based on previous mechanical testing experiments^32^ and subsequently stretched until failure at a displacement rate of 20 mm/min and a sampling rate of 100 Hz. The surface of the tested samples was recorded with a single charge-coupled camera (Q400; 2.8 megapixels; Limess, Krefeld, Germany), which was oriented perpendicular to the sample surface to enable in-plane digital image correlation (DIC). ISTRA 4D software (VRS 4.4.1.354; Dantec Dynamics, Ulm, Germany) was used to calculate the engineering strain by a virtual extensometer within the gauge length of each sample. Both ITB and DM samples were stretched according to the alignment of the main collagen fiber orientation. Strain data for the DM samples used DIC measurements while the ITB samples used crosshead displacement measurements (Fig. 2).

### Water content analysis

**Fig. 3:**
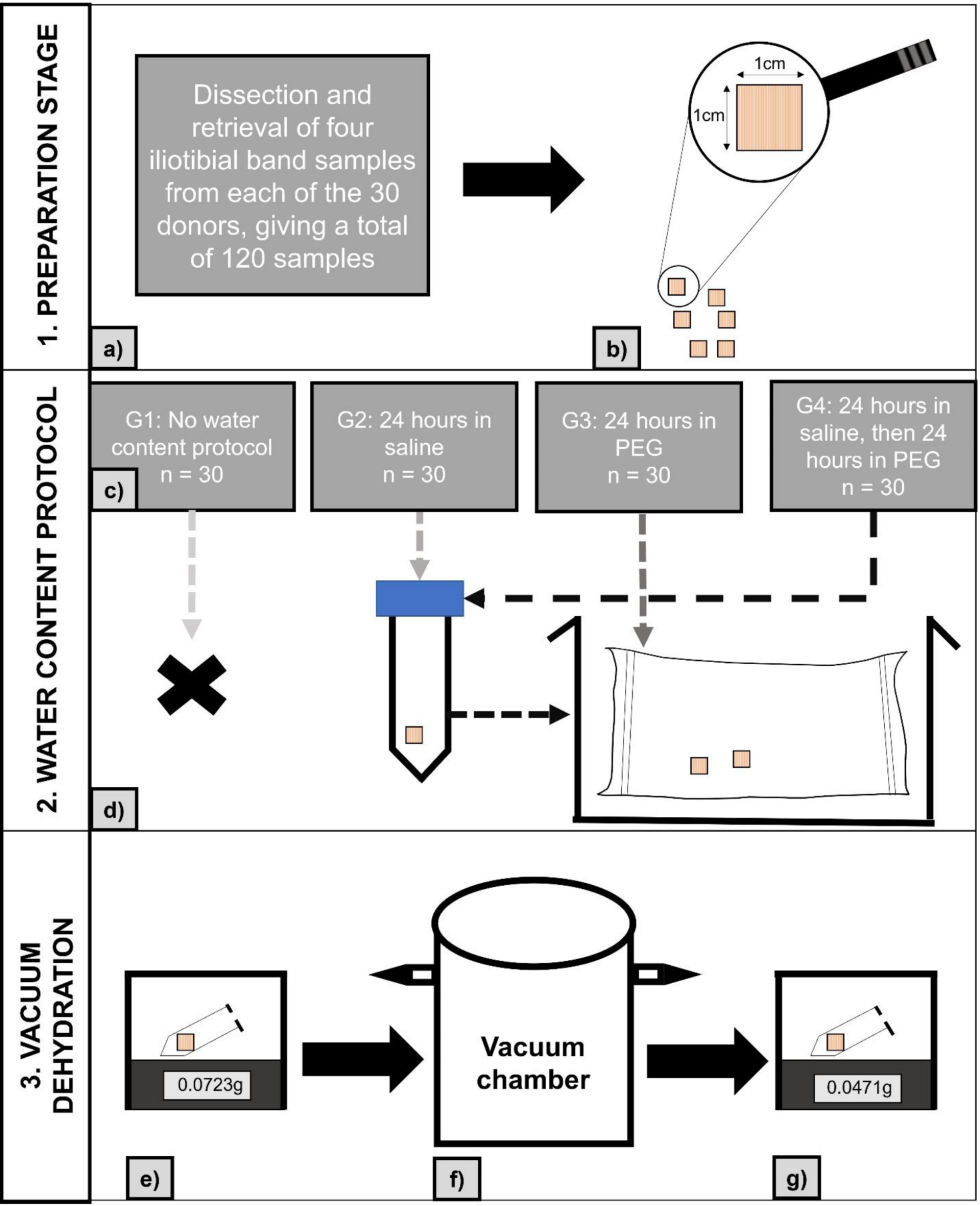
Diagram demonstrating the methodology to prepare samples for water content analysis in different groups (G1, G2, G3, G4). 1: Preparation stage, (**a**) sample retrieved from donors, (**b**) samples were sectioned into comparable 1 cm x 1 cm pieces. 2: Water content protocol, (**c**) samples from each donor were allocated across groups, (**d**) Samples placed in water content adjustment conditions. 3: Vacuum dehydration, (**e**) wet sample weighed, (**f**) sample placed in vacuum chamber to vaporise water overnight, (**g**) dry sample weighed. Given weights in step 3 are only examples.

120 ITB samples measuring 1 cm x 1 cm were retrieved from a cohort of 30 Crosado-embalmed cadavers (85 ± 10 years; 10 females, 20 males) including but not limited to the ones used for biomechanical testing (Fig. 3, step 1). Samples from each cadaver were then divided into four groups of 30 samples (Fig. 3, step 2): (1) untreated prior to testing, (2) 24 h in saline solution (0.9% sodium chloride in water), (3) 24 h within a sealed dialysis membrane (Spectra/ Por^®^), within 2.5% PEG by weight, (4) 24 h in saline solution, followed by 24 h in a dialysis membrane within 2.5% PEG. The water content of the tissues was then measured by vacuum dehydration, allowing the water within the tissue to be placed under vacuum pressure so that it vaporises (Fig. 3, step 3).

### Data processing and statistical analysis

The E_mod_, UTS, F_max_, and strain at maximum force (SF_max_) data were calculated from the force readings and synchronized strain data using MATLAB R2017b software (Mathworks, Natick, MA, USA). As a backup, the respective biomechanical properties were also available from the crosshead displacement data of the machine. For statistical analyses, Excel version 16.65 (Microsoft Corporation, Redmond, WA, USA) and GraphPad Prism version 9 (GraphPad Software, La Jolla, CA, USA) were used. The data were assessed graphically, and a Shapiro-Wilk test was applied to determine whether the data were normally distributed. Normally distributed data were analyzed using one-way ANOVA including Tukey correction for multiple comparisons of the ITB UTS (fresh-frozen PEG only, Crosado-embalmed saline then PEG, and Crosado-embalmed PEG only). For non-normally distributed data, the Kruskal-Wallis test including Dunn’s correction for multiple comparisons was applied. The Mann-Whitney-U test was used to compare non-parametric unpaired groups. An independent t-test was used for unpaired parametric data. P-values equal to or smaller than 0.050 were considered statistically significant. Values are given means ± standard deviation unless specified otherwise.

## Results

### Biomechanical properties of fresh crosado-embalmed ITB and DM samples

For the ITB, the E_mod_ of all tested groups were significantly different from each other with fresh samples (110 MPa, 49.2–160.1 MPa; median, range) showing the lowest values and Crosado-embalmed samples only treated with PEG (295 MPa, 170.4–512.2 MPa) having the highest values. The UTS of Crosado-embalmed ITB samples (46 ± 15 MPa, PEG only; 37 ± 13 MPa, saline then PEG) was higher compared to fresh samples (21 ± 8 MPa), while the SF_max_ significantly differed only for between fresh (25%, 6.6–29.6%) and Crosado-embalmed (20%, 13.1–24.1%) ITB samples only treated with PEG (*p* = 0.01). The F_max_ revealed no statistically significant differences for any of the comparisons for the fresh and Crosado-embalmed ITB.

**Fig. 4:**
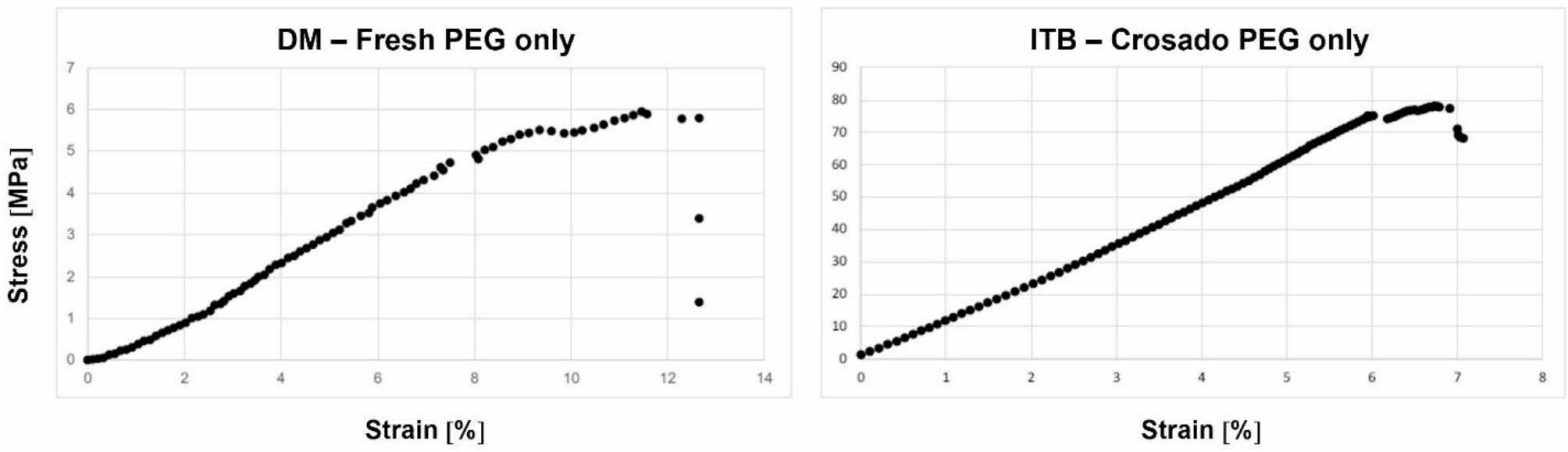
Exemplary stress-strain curves of a fresh iliotibial band (ITB) sample and a Crosado-embalmed dura mater (DM) sample are depicted. Both samples were treated with polyethylene glycol (PEG) only.

For the DM, the Crosado-embalmed samples revealed significantly higher E_mod_, UTS and F_max_ values compared to fresh samples. An exemplary stress-strain curve of the ITB and DM both treated with PEG is represented in Fig. 4.

The means, medians, and standard deviations for all here analysed biomechanical properties including significant *p*-values of the statistical comparisons between the groups are depicted in Table 2.

**Table 2:** The biomechanical properties (median in parentheses) and *p*-values of the statistical comparisons are depicted for the iliotibial band (ITB) and the dura mater (DM).

	Biomechanical properties					Statistical comparisons			
	ITB			DM		ITB			DM
	1 Fresh PEG only	2 Crosado PEG only	3 Crosado saline then PEG	4 Fresh PEG only	5 Crosado PEG only	1 vs. 2	1 vs. 3	2 vs. 3	4 vs. 5
F_max_	120 ± 55 (120)	151 ± 57 (137)	184 ± 115 (142)	21 ± 20 (17)	25 ± 13 (20)	ns	ns	ns	0.050
E_mod_	108 ± 31 (110)	306 ± 91 (295)	202 ± 75 (184)	69 ± 44 (53)	154 ± 130 (117)	< 0.001	< 0.001	0.041	< 0.001
UTS	21 ± 8 (22)	46 ± 15 (49)	37 ± 13 (35)	7 ± 4 (5)	18 ± 12 (16)	< 0.001	0.001	ns	< 0.001
SF_max_	24 ± 6 (25)	20 ± 4 (20)	21 ± 5 (21)	11 ± 3 (11)	11 ± 7 (10)	0.010	ns	ns	ns

E_mod_, elastic modulus [in MPa]; F_max_, maximum force [in N]; UTS, ultimate tensile strength [in MPa]; SF_max_, strain at maximum force [in %]; ns, not significant (*p* > 0.050).

### Cross-sectional areas of tested ITB and DM samples

For both the ITB and DM, the CSA of fresh samples following osmotic pressure protocols was significantly greater than the one for Crosado-embalmed samples submerged in PEG only. The means, medians, and standard deviations for the CSAs of the ITB and the DM including the *p*-values of the respective intra-tissue comparisons are shown in Table 3.

**Table 3:** The cross-sectional areas [in mm^2^] (mean ± standard deviation, median in parentheses) and p-values of the statistical comparisons are depicted.

Cross-sectional areas					Statistical comparisons			
IBT			DM		IBT			DM
1 Fresh PEG only	2 Crosado PEG only	3 Crosado saline then PEG	4 Fresh PEG only	5 Crosado PEG only	1 vs. 2	1 vs. 3	2 vs. 3	4 vs. 5
5.8 ± 2.1 (5.6)	3.4 ± 1.2 (3.4)	5.0 ± 2.6 (4.1)	3.1 ± 1.2 (2.9)	1.1 ± 0.5 (1.0)	0.003	ns	ns	< 0.001

Ns, not significant (p-value > 0.050). CSA, cross-sectional area; DM, dura mater; ITB, iliotibial band; PEG, polyethylene glycol.

### Water content analysis of Crosado-embalmed ITB samples

Water content analysis showed that 24 h saline, 24 h PEG, and 24 h in saline followed by 24 h in PEG yielded significantly higher water content than untreated samples. Significantly greater water content was present in samples of the 24-hour PEG group and 24-hour saline followed by 24-hour PEG group, when compared to 24 h in saline alone. The means, medians and standard deviations of the water content of the Crosado-embalmed ITB samples and the p-values of the respective group comparisons are shown in Table 4.

**Table 4:** Descriptive data (mean ± standard deviation, median in parentheses) and significant p-values of the water content [in %] analyses are shown for the human crosado-embalmed iliotibial band.

ITB water content				Statistical comparisons					
1 Untreated	2 Saline 24 h	3 PEG 24 h	4 Saline 24 h then PEG 24 h	1 vs. 2	1 vs. 3	1 vs. 4	2 vs. 3	2 vs. 4	3 vs. 4
49 ± 7 (48)	73 ± 4 (73)	77 ± 4 (77)	78 ± 4 (78)	< 0.001	< 0.001	< 0.001	0.003	< 0.001	ns

Ns, not significant (p-value > 0.050). PEG, polyethylene glycol.

## Discussion

Both the ITB and the DM predominantly consist of collagen within the extracellular matrix, alongside a broad variety of unformed extracellular matrix components including proteoglycans and glycoproteins to varying extent^28,33–37^. Moreover, previous studies on collagen-rich tissues revealed a negligible contribution of cells to the overall load-bearing capacity^38,39^. Hence, the biomechanical properties and three-dimensional structure of both tissues is considered closely related to its collagen backbone. The water content of tissues contribute to influencing the biomechanical properties and maintaining the collagen fibril network, along with glycosaminoglycans^40^. Water also is important in collagen fibril functioning as it plays a role in glycation, particularly during ageing, as water has an affinity to advanced glycation end products in collagen fibrils^41^. Collagen and proteoglycan scaffolds are thought to maintain the osmotic pressure^42^, along with cells within tissues^28^.

Osmotically adaption prior to biomechanical testing is a routine step for fresh tissues to reach a similar hydration state of all samples for unifying the starting conditions^23,25^. Several biomechanical properties of soft tissues are dependent on hydration^26^, and avoiding both hyper- and hypohydration results in more homogeneous data sets. Commonly, the osmotic stress protocol involves submerging the samples within a dialysis membrane in a polyethylene glycol (PEG) solution of a defined concentration for a certain amount of time prior to the test^27^. This process should be individually established for each tissue type^27^. For the fresh human ITB and DM, immersion in PEG solution with a concentration of 2.5%^28^ and 5%^23,25^ for 24 h is considered to provide the most natively representative results. For Crosado-embalmed samples, an additional rinsing step in normal saline solution (0.9% sodium chloride) is considered beneficial in attempt to wash out the excess stiffening components, such as ethanol and glycerine^29^, thereby recovering the tissue as close as possible to its native mechanical behaviour. Furthermore, the water content of Crosado-embalmed samples including the influence of fluids used for rinsing and osmotic adaptation should be analysed together with the CSA of the mechanically tested samples to determine the impact on the geometry of such tissues.

### Crosado-based embalming alters the cross-sectional area of the tested samples

The mechanically tested samples of both tissue types revealed lower CSAs for Crosado-embalmed specimens when compared to fresh samples when uniformly treated with PEG for 24 h prior to the test. An additional 24-hour saline treatment prior to the PEG treatment of Crosado-embalmed ITB samples increased the CSAs, bringing them closer to fresh tissues following osmotic adaption alone. Water content analyses demonstrated that untreated Crosado-embalmed ITB samples with a mean value of 49% water content stayed far below the mean fresh ITB water content of 69%, which was established previously^28^. For Crosado-embalmed samples, which were treated with a protocol of 24-hours in saline only, a significantly raised water content of 74% was measured, which again was significantly lower compared to an additional PEG treatment, or PEG treatment only, with 78% and 77%, respectively. The hyperhydration of Crosado-embalmed tissues after applying the identical osmotic protocol as for fresh samples infers that the PEG and saline solution are likely hypotonic compared to Crosado-embalmed tissues. Following the treatment with the Crosado embalming, the tissues contain additional osmotically active agents such as dimethyl ammonium chloride. This likely results in greater uptake of water into the tissues’ extracellular matrix or hypertonic fibroblast cells than native tissues and might irreversibly distort the three-dimensional structure of the tissue due to the fact that these agents cannot be washed out properly.

Combining the CSA and water content results of the ITB, it can be shown that a higher CSA after osmotic adaptation of one sample group compared to another does not imply a concomitant higher water content. Even though fresh ITB samples revealed significantly higher CSA values compared to equally osmotically adapted Crosado-embalmed samples, their mean water content by weight was lower^28^. From this, two potential conclusions may be drawn: Firstly, Crosado-based embalming appears to alter the tissues’ ability to store water. Secondly, the significantly lower CSA of Crosado-embalmed ITBs cannot be attributed to its altered hydration alone but is likely caused by fixative-induced alterations of the extracellular matrix proteins, predominantly collagen. It is generally accepted that formaldehyde as a component of the Crosado fixative^21^ cross-links collagen^43^. The remaining components of the embalming and moistening solution of the Crosado fixative are ethanol, glycerine dimethyl ammonium chloride, and water^21^. cross-links collagen^43^. The remaining components of the embalming and moistening solution of the Crosado fixative are ethanol, glycerine dimethyl ammonium chloride, and water^21^. Ethanol and glycerol within glycerine have a dehydrating effect on soft tissues^29^ and, therefore, cause the lower water content of Crosado-embalmed tissues compared to fresh tissues plausibly. The solvent phenoxyethanol was shown to potentially hamper the ability of water to wash formaldehyde out of biological tissues^44^. Thereby, it likely contributes to the here shown higher E_mod_ due to formaldehyde cross-linking effects onto the extracellular matrix. Dimethyl ammonium chloride has antibacterial effects^45^ with unknown but likely negligible effects to the collagen backbone of the here tested samples. However, the role of these components requires further study to elucidate their significance, and determine the resulting combined effects on tissue dehydration, cross linking and chemical component wash-out.

### The load-bearing capacity of the tested samples seems to be unaffected by the Crosado fixative

For both the ITB and the DM, collagen is a main protein of the extracellular matrix^33,34,46^. Hence, if embalming induces collagen damage this would be associated with a significant decrease in UTS values of Crosado-embalmed samples compared to fresh samples. However, care must be taken when interpreting the CSA-related biomechanical properties such as the E_mod_ and UTS given the effects of the embalming on the CSA described in the previous discussion section. As all samples in this study were trimmed to an identical 2D dumbbell shape before the mechanical test, the F_max_ values are of value when assessing their load-bearing capacity.

For both ITB and DM, the F_max_ values of Crosado-embalmed samples were similar to fresh samples. Thus, from a mechanical point of view, there is no evidence to date of a significant disintegration of the collagen backbone through the Crosado fixative. The higher F_max_ values of Crosado-embalmed samples compared to fresh samples were different only for DM samples. Higher F_max_ values of Crosado-embalmed DM samples are explicable through the timing when the samples were cut in relation to the osmotic treatment. Here, as per standard protocol, all fresh and embalmed mechanically tested samples were cut before submerging them into the hydrating solutions. In this study, Crosado-embalmed samples were shown to be hypertonic compared to fresh samples. It may be hypothesised that the changes in CSA result from macroscopic and molecular alterations related to a non-destructive alteration of the collagen backbone potentially affecting its water-binding capacity. Further work is required to determine the morphological and chemical effects of Crosado-embalming, using techniques such as histology, spectroscopy, or x-ray diffraction. Specifically, this would aid in determining if the number of collagen fibres differs between equal-sized samples retrieved from both Crosado-embalmed samples and fresh samples prior to osmotic stress treatment and to aid in explaining the herein increased F_max_ values when stretched to failure. Alternatively, or perhaps even in addition to the given explanation, collagen cross-linking through formaldehyde might cause higher F_max_ values in samples treated with the Crosado fixative, as shown for DM. In histology, it is common practice to break cross-links using alkaline or chelating agents. Future studies should investigate the effect of such agents on the biomechanical properties of soft tissues, which were treated with formaldehyde or formaldehyde containing fixatives.

### Cross-sectional area appears a poor predictor of water content

The results of the water content analyses yielded that an additional 24-hour saline treatment before the 24-hour osmotic adaptation in PEG did not alter the water content of the ITB (77 ± 4% vs. 78 ± 4%). Further to this, the CSAs of samples with the additional 24-hour step in saline (5.0 ± 2.6 mm^2^) were statistically similar to fresh samples (5.8 ± 2.1 mm^2^). In contrast, the CSA of samples with PEG treatment only (3.4 ± 1.2 mm^2^) was significantly lower compared to fresh samples. Previous research has shown that salts, such as NaCl found in saline, have a negligible effect on the biomechanical properties of collagen^47^, despite other researchers noting that the ionic reactions might cause reduction of collagen entanglements^48^. Therefore, it is likely that the additional saline step causes swelling of cells, as each Na + and Cl- bind to a total of 6 water molecules. This distortion of cells situated within the extracellular matrix, like decellularization, may result in extracellular matrix damage and have a considerable impact on the CSA of the tested sample. As such, hydration is one influencing factor of the CSA of the tissues however, there are likely many influencing factors and as such CSA measurements cannot be reliably utilised to predict biomechanical properties.

### There are limitations to the use of Crosado-embalmed samples for uniaxial biomechanical studies

This study revealed that the Crosado fixative causes alterations to the CSA of collagen-rich human soft tissues compared to the native state, which appears to persist using PEG and normal saline at given concentrations and submerging times. Osmotic protocols with saline followed by PEG appears to potentially alter the morphological properties of tissues. Therefore, using the protocol applied in this study, Crosado-embalmed tissues cannot be used as a suitable homologue to investigate the native uniaxial biomechanical properties of the human ITB and DM for computational or physical human models. However, as the CSA alterations are rather predictable, Crosado-embalmed tissues seem well suited for preliminary uniaxial biomechanical investigations to understand the impact of other extraneous variables, especially if access to native (human) biomaterial is limited. Moreover, previous studies using Crosado-embalmed tissues provided comprehensible results on fundamental biomechanical queries which still hold great value^3–5^. In summary, fresh human samples are preferable for biomechanical testing when available.

### Limitations

This study is limited in its sample size, and as such uses data from various subgroups. For the tissue preparation and the mechanical testing procedure, the stated limitations of previous studies apply here also^22,23,25,49–51^. Furthermore, the different mean ages and sex ratios of the subgroups might have impacted the results, however tissues from the same cadavers were used across groups wherever possible. Additionally, errors relative to the measurement inaccuracy, such as the load cell or other aspects of the testing set up, may have impacted the results. For the water content analysis, although considered negligible, the scale of this analysis was limited by physical and logistical restrictions, e.g., tissues availability and storage, laboratory humidity and temperature, and time to process, therefore if investigated in a different environmental context the results may differ. In this work, the biomechanical effects of the Crosado embalming on DM and ITB are predominantly explained through its effects on collagen as the main protein of the extracellular matrix. However, the Crosado fixative might also affect other extracellular proteins and cells. Due to technical issues, DIC-based data was only available for the DM. The here reported properties of the ITB were based on the crosshead-displacement data, which might have affected the stated results through stiffness, tailoring influences and deviations of the calculated strains compared to an optical approach in the parallel length of the samples. The predominant collagen fibre orientation was assessed based on the orientation of collagen fibres on the surface of the sample, which is likely an oversimplification when assessing the collagen orientation of the overall multi-layered sample. Here, all mechanically tested samples were sectioned prior to the rehydration treatment utilizing a standardized cutting template, and their CSAs were promptly determined just before the commencement of the mechanical test, aligning with the methodology established in previous investigations^23,25,38,39,51^. This approach was adopted to maintain a consistent number of collagen units within both the gauge length and width, thereby ensuring homogeneity. Consequently, any collagen degradation induced by the hydration treatment would manifest mechanically through notable alterations in F_max_ and UTS. Alternatively, the sample sectioning process could have been performed post-rehydration, resulting in uniform sample sizes but potentially introducing variations in collagen content within the gauge length area of the specimen. Finally, only ITB but not DM samples were used for water content analysis, due to the limited availability of tissues. In this study, the biomechanical properties under quasi-static loading along the direction of main fibre bundle orientation were analysed. Different loading velocities and directions should be explored in future studies.

## Conclusion

Crosado-based embalming alters the cross-sectional area and biomechanical properties of the human ITB and DM samples compared to native samples. These observations may be related to deformations in the collagen backbone and its water-binding capacity. Thereby, there was a marked increase of biomechanical properties in collagen-rich samples that were calculated using cross-sectional area values (E_mod_ and UTS), while simultaneously the loading capacity remains largely unaltered when compared to fresh samples. The CSA alone appears an inappropriate measure for the water content, as water contributes to cell swelling and cross-linking should be considered.

## Data Availability

Data is available upon request from the corresponding authors.
